# Risk factors and outcome of *Pseudomonas aeruginosa* bloodstream infections (PABSI) in hematological patients: a single center retrospective cohort study

**DOI:** 10.1007/s15010-024-02453-0

**Published:** 2024-12-19

**Authors:** Johanna Kessel, Gesine Bug, Björn Steffen, Uta Brunnberg, Maria J.G.T. Vehreschild, Sarah Weber, Sebastian Scheich, Fabian Lang, Hubert Serve, Eva Herrmann, Michael Hogardt

**Affiliations:** 1 Department of Internal Medicine 2, Infectious Diseases, Goethe University Frankfurt, University Hospital Frankfurt, Theodor Stern-Kai 7, 60590 Frankfurt, Germany; 2 Department of Medicine 2, Hematology/Oncology, Goethe University Frankfurt, University Hospital Frankfurt, Theodor Stern-Kai 7, 60590 Frankfurt, Germany; 3 Institute of Biostatistics and Mathematical Modelling, Goethe University Frankfurt, Frankfurt, Germany; 4 Institute of Medical Microbiology and Infection Control, University Hospital Frankfurt, Goethe University, Frankfurt, Germany

**Keywords:** *Pseudomonas aeruginosa*, Bloodstream infections, Antibiotic resistance, Fluoroquinoloneprophylaxis, Hematological patients, Antimicrobial stewardship

## Abstract

**Purpose:**

Bloodstream infections caused by *Pseudomonas aeruginosa* (PABSI) in hematological patients are associated with high morbidity and mortality. We investigated the epidemiology, risk factors, and outcomes of PABSI at our center.

**Methods:**

All adult hematological patients with PABSI between January 2013 and July 2023 were included. Demographic and clinical characteristics, antimicrobial susceptibilities, antibiotic therapy, fluoroquinolone-prophylaxis, source of infection, and 30-day outcome were recorded. Descriptive statistics, tests for difference, and logistic regression models were performed.

**Results:**

Fifty patients with PABSI were identified with a median age of 58.5 years (range 24–78). 37 patients (74%) had severe neutropenia, 20 (40%) received allogeneic HSCT, and 29 (58%) had acute leukemia. A total of 34 (68%) had received timely appropriate anti-pseudomonal antibiotic therapy. The most common presumed cause of PABSI was mucositis (*n* = 16, 32%), followed by pneumonia (8, 16%) and skin and soft tissue infections (*n* = 6, 12%). Empirical combination therapy was used in 16 (32%) patients, while targeted combination therapies were used in 27 (54%) patients. *P. aeruginosa* detection led to treatment change in 31 (62%) cases. The overall 30-day survival rate was 78% (*n* = 39). Carbapenem-resistance occurred in 34% (*n* = 17), and multidrug-resistance (MDR) in 20% (*n* = 10). Prior antibiotic exposure was associated with resistance. Appropriate antibiotic therapy was associated with survival, whereas antibiotic resistance and organ infection were associated with a fatal outcome.

**Conclusion:**

Prior antibiotic exposure in hematological patients is associated with resistance in PABSI, which is a major risk factor for a fatal outcome. Antibiotic stewardship efforts should be intensified and fluoroquinolone prophylaxis needs to be reconsidered.

## Introduction

Bloodstream infections (BSI) caused by gram-negative bacteria are of particular concern in hematological patients. *Pseudomonas aeruginosa* is a major threat, due to its inherited antibiotic resistance. *P. aeruginosa*-associated bloodstream infections (PABSI) in particular are associated with high morbidity and mortality [1, 2]. The unique characteristics of hematological inpatients include a compromised immune system, a mucosal barrier disturbance due to mucositis, central venous catheters and frequent exposure to health care settings, making them particularly susceptible to nosocomial infections including PABSI. In particular in hematological patients, we previously demonstrated that BSIs caused by non-fermenters including *P. aeruginosa* are associated with a worse outcome compared to *Enterobacterales* BSIs [3]. Antibiotic therapy with a beta-lactam antibiotic with anti-pseudomonal activity is strongly recommended in febrile neutropenia to adequately cover BSI caused by *P. aeruginosa* [4]. Sources of PABSI are sometimes found in the inpatient environment, such as hospital water systems [5]. *P. aeruginosa* can cause a variety of infectious diseases, including pneumonia, skin and soft tissue infections, wound infections and urinary tract infections, which can lead to BSI through bacterial translocation or in the context of central venous catheter-associated infections [6, 7]. It has been demonstrated that infections caused by multidrug-resistant (MDR) *P. aeruginosa* are associated with prior quinolone use and previous hospitalization, particularly at intensive care units [8]. In a large, multicentric, retrospective cohort study, fluoroquinolone prophylaxis and prior therapy with carbapenems or piperacillin/tazobactam were identified as risk factors for bloodstream infections by MDR *P. aeruginosa*, as well as previous therapies with *Pseudomonas* cephalosporins and PABSI occurring under fluoroquinolone prophylaxis [9, 10]. These risk factors often apply to hematological patients. PABSI due to MDR *P. aeruginosa* are more frequently treated inadequately and are associated with increased mortality [11–13]. Understanding the risk factors and outcome of PABSI in this patient population is crucial for optimizing patient management and improving outcomes.

## Methods

A retrospective study was conducted at the hematological department (67 beds) of the University Hospital Frankfurt. The medical records of all adult patients admitted and treated on hematology wards for a malignant hematological disease or myeloproliferative disorder, and diagnosed with PABSI between January 2013 and July 2023, were reviewed. Data on patient demographics, clinical characteristics, the results of microbiological susceptibility tests, antibiotic therapy and previous antibiotic use, the potential source of infection and patient outcomes at day 30 after detection of BSI were recorded. Patients were treated according to local guidelines when febrile neutropenia occurred. First-line therapy was piperacillin/tazobactam, imipenem for colonization with ESBL-producers. In sepsis and colonization with carbapenem-resistant *Enterobacterales* or MDR *P. aeruginosa*, (recommendation before new β-lactam-β-lactamase inhibitor combinations or cefiderocole were available) high-dose meropenem was used as a prolonged infusion plus colistin in accordance with local guidelines. If BSI due to a susceptible pathogen is proven, colistin would be immediately discontinued, as well as in cases, where no pathogen has been detected, but patients´ condition improved. In cases of catheter infection, grade III or IV mucositis or the presence of a soft tissue infection, a glycopeptide or linezolid would also be prescribed. The procedure regarding levofloxacin prophylaxis (Levo-P) was inconsistent: All hematological patients outside the stem cell transplant unit received Levo-P during neutropenia grade IV. In our stem cell transplant unit, Levo-P was discontinued in 2018, only to be re-established as standard in 2020 during the Covid-19 pandemic and discontinued again in 2022/2023. Instead, when febrile neutropenia occurred at night, meropenem was given after sampling of blood cultures. Targeted therapy or empirically de-escalated therapy was initiated in the event of clinical stabilisation with no evidence of infection by MDR pathogens. This de-escalation strategy is predominantly pursued in our clinic.

We also analyzed the local epidemiology of *P. aeruginosa* during the observation period with regard to multidrug resistance.

Species identification and antibiotic susceptibility testing was performed as previously described [14]. Of note, phenotypic antibiotic susceptibility testing was performed until 2018 according to Clinical and Laboratory Standards Institute (CLSI) guidelines and since 2019 according to EUCAST.

Data was processed and analyzed using SPSS (IBM SPSS version 29). Univariate comparisons were conducted by using the *x*^*2*^ or the Fisher exact test for categorical variables. Tests for normal distribution were performed using the Kolmogorov Smirnov test. For non-normal distributed data, the Mann-Whitney U estimator was used. Variables with a *P* value < 0.05 in univariate regression analysis could be included in the multivariate regression analysis (binary logistic regression). All tests were two-tailed, with the significance level set at 0.05. The study was approved by the local ethics committee (#2021 − 370).

### Definitions

The onset of BSI was defined as the date of collection of the positive blood culture sample. Severe neutropenia (grade IV) was defined as an absolute neutrophil count of less than 0.5 × 10^9^ cells/liter. Carbapenem resistance was defined as either imipenem and/or meropenem resistance, fluoroquinolone resistance was defined as levofloxacin and/or ciprofloxacin resistance according to the respective available EUCAST definitions. The “I” category was interpreted as resistant in all isolates tested as “intermediate” prior to January 2020 (CLSI) and interpreted as susceptible in all isolates tested “susceptible increased exposure” from first January 2020 (EUCAST) [15, 16]. The term “multidrug resistance” was defined as resistances in three or more of the following antimicrobial classes, namely penicillins (piperacillin/tazobactam), cephalosporins (ceftazidime or cefepime), carbapenems (imipenem or meropenem), and fluoroquinolones (ciprofloxacin). Empirical antibiotic therapy was defined as any antibiotic treatment administered for suspected BSI prior to the availability of definite susceptibility results from blood cultures. Any antibiotic treatment initiated following the availability of susceptibility results was defined as targeted therapy. *Pseudomonas*-directed monotherapy was defined as the administration of one in vitro active anti-pseudomonal agent, whereas the administration of two or more in vitro active antibiotics was defined as combination therapy. Therapy extension was defined as either a change to combination therapy from monotherapy, or the use of any of the new beta-lactam/beta-lactamase inhibitor combinations ceftazidime/avibactam, ceftolozane/tazobactam, imipenem/relebactam, or cefiderocol, or colistin. Appropriate therapy was defined as the use of one or more *Pseudomonas*-active agents, tested as susceptible in vitro. Antibiotic pre-exposure was defined as prescription of the respective antibiotic drug for at least 48 h within the last three months before the diagnosis of PABSI. Acute kidney injury was defined according to the KDIGO criteria as an increase in serum creatinine ≥0,3 mg/dl within 48 h or an increase in serum creatinine to at least 1.5 times the known or assumed baseline value within seven days or a drop in urine volume to < 0.5 ml/kg bw/h for at least six hours [17]. Cases receiving an allogeneic stem cell transplantation (SCT) included patients hospitalized prior to or after having received conditioning chemotherapy and up to day 100 after SCT.

## Results

A total of 50 patients were included into the analysis, with a median age of 58.5 years and a slight male predominance (64% were male). The majority of patients (*n* = 25, 50%) had acute myeloid leukemia (AML), (*n* = 8, 16%) acute lymphoblastic leukemia (ALL), and (*n* = 8, 16%) non-Hodgkin lymphoma. Among 29 patients (58%) who underwent hematopoietic stem cell transplantation (HSCT), 20 (40%) underwent allogeneic HSCT. Most patients were in a good (ECOG 0,28%) or slightly reduced (ECOG 1, 38%) performance status at the time of admission. A total of 11 patients (22%) died within 30 days after onset of PABSI. Of these, 10 deaths could be defined as sepsis-associated. In one case, the cause of death could not be determined; in this case too, the most likely cause was sepsis. Of the eleven sepsis patients, five died within 72 h of the blood cultures being taken. Eight patients fulfilled the criteria for septic shock [18]. All of them died within 30 days, five of them within 72 h of the onset of PABSI. The patients of our cohort died after a median of 7 (2–30) days. The most common suspected sources of PABSI were mucositis (*n* = 16, 32%), skin and soft tissue infections (*n* = 10, 20%), among those four perianal abscesses (8%), further pneumonia (*n* = 8, 16%), and urinary tract infections (*n* = 5, 10%). Additionally, catheter-related infections and complicated intraabdominal infections were observed in three cases each (6% each). One case of malignant otitis externa was identified. In five cases (10%), no potential source of infection could be identified, four patients (8%) had more than one potential source of infection. In most cases (*n* = 45, 90%), fever triggered the collection of blood cultures, which then provided evidence of PABSI. In five cases, deterioration of the general condition and/or increased inflammation values led to the sampling of blood cultures (Table 1).

**Table 1:** Patient characteristics

Characteristics		All patients (*n* = 50)
Age (years; median, range)		58.5 (24–78)
Female sex, n (%)		18 (36)
BMI (median; range)		24.5 (16.8–45.5)
Hematological disease, n (%)	Acute leukemia	33 (66)
	Chronic leukemia	3 (6)
	Lymphoma	9 (18)
	Other	5 (10)
HSCT, n (%)	No	21 (42)
	Yes	29 (58)
	Allogeneic SCT < 100d	20 (40)
	Autologous SCT	2 (4)
	Allogeneic SCT > 100d	7 (14)
ECOG, n (%)	0	14 (28)
	1	19 (38)
	2	5 (10)
	n.a.	12 (24)
Reason for BC sampling, n (%)	Fever	45 (90)
	Suspected systemic infection without fever	5 (10)
Neutropenia grade IV		39 (78)
Duration of neutropenia grade IV after onset of PABSI(days; median, range)		7 (0–89)
LOS prior to PABSI days, median (range)		12.5 (0–74)
Length of stay (days; median, range)		30 (1-149)
Suspected source of PABSI, n (%)	Mucositis	16 (32)
	SSTI including perianal abscess	10 (20)
	Pneumonia	8 (16)
	UTI	5 (10)
	Intraabdominal infection	3 (6)
	Catheter-related	3 (6)
	Otitis externa	1 (2)
Empirical antibiotic therapy, n (%)	Combination therapy	16 (32)
	Monotherapy	34 (68)
Regimen containing agents, n (%)	Piperacillin/Tazobactam	17 (34)
	Meropenem	22 (44)
	Imipenem	9 (18)
	Levofloxacin	3 (6)
	Ciprofloxacin	5 (10)
	Ceftazidime/Avibactam	1 (2)
	Amikacin	6 (12)
	Tobramycin	1 (2)
	Colistin	4 (8)
Targeted antibiotic therapy, n (%)	Combination	33 (66)
	Extension	31 (62)
	Step-down	4 (8)
30-day mortality, n (%)		11 (22)
Septic shock, n (%)		8 (16)
Death (sepsis-related) n (%)		10 (20)

One-third of the patients (*n* = 16, 32%) received an empirical anti-pseudomonal combination therapy. Meropenem was the most frequently prescribed antibiotic (*n* = 22, 44%), followed by piperacillin/tazobactam (*n* = 17, 34%) and imipenem (*n* = 9, 18%). In one case, a non- *P. aeruginosa*-active regimen with ceftriaxone was initiated. Fluoroquinolones were added in seven cases (14%) as part of a combination therapy, and in one case prescribed as an empirical monotherapy, aminoglycosides were only prescribed as combination partners to anti-pseudomonal beta lactam-antibiotics. Four patients (8%) were treated with colistin as part of a combination therapy, and in one case (2%), ceftazidime/avibactam was prescribed on an empirical basis for a known colonization with MDR *P. aeruginosa.* In 37 (74%) cases, the detection of PABSI led to a treatment modification. Two-thirds (*n* = 33, 66%) received targeted anti-pseudomonal combination therapy, in 31 cases (62%) a treatment extension (broader spectrum agent and/or combination therapy) was carried out.

To identify potential risk factors for fatal outcome, we conducted a comparison of the characteristics of 30-day survivors and non-survivors.

Appropriate empirical therapy within 24 h was significantly more frequent in cases with a favorable outcome. There was no difference in survival if either empirical or targeted combination therapy was administered. There were also no differences regarding previous *Pseudomonas*-active antibiotic therapies and prophylaxis. Patients with infections of internal organs (lung or intraabdominal infection) had a significantly worse outcome (*p* < 0.001) compared to cases with bacterial translocation favored by mucositis, or cases with UTI or other sources of PABSI (Table 2). Acute kidney injury according to KDIGO criteria was also significantly more frequent in cases with fatal outcome. Resistant *Pseudomonas* isolates were more common in patients who had a poor 30-day outcome.

**Table 2:** Risk factors of fatal outcome at day 30 in PABSI

Characteristics		Overall *N* = 50 (%)	Survivors *N* = 39 (%)	Non-survivors *N* = 11 (%)	*p*-value^1^
Appropriate antibiotic therapy within 24 h		35 (70)	31 (79)	4 (36)	**0.01**
Empirical combination therapy		16 (32)	13 (33)	3 (28)	1.0
Change to modified targeted therapy		37 (74)	28 (72)	9 (82)	0.70
	Combination	33 (66)	27 (69)	6 (55)	0.36
	Extension	31 (62)	23 (59)	8 (73)	0.50
	Step-down	4 (8)	4 (10)	0 (0)	0.56
Suspected source of PABSI	Pneumonia	8 (16)	4 (10)	4 (36)	0.059
	Perianal abscess	4 (8)	4 (10)	0 (0)	0.56
	Mucositis	16 (32)	14 (36)	2 (18)	0.47
	Catheter-related	3 (6)	3 (8)	0 (0)	1.0
	SSTI	6 (12)	4 (10)	2 (18)	0.60
	UTI	5 (10)	5 (13)	0 (0)	0.57
	Otitis externa	1 (2)	1 (3)	0 (0)	1.0
	Intraabdominal infection	3 (6)	0 (0)	3 (28)	0.008
	Internal organ infection (Pneumonia or cIAI)		4 (10)	7 (64)	**< 0.001**
Septic shock		8 (16)	0 (0)	8 (73)	**< 0.001**
Neutropenia grade IV		38 (76)	30 (77)	8 (73)	1.0
Duration of neutropenia > 21d prior PABSI		11 (22)	6 (15)	5 (45)	**0.048**
LOS prior to PABSI d, median (range)		12.5 (0–74)	13 (1–74)	7 (0.61)	0.82
Acute kidney injury		15 (30)	4 (10)	11 (100)	**< 0.001**
PA-active antibiotic therapy within 3 months prior PABSI		36 (72)	28 (72)	8 (73)	1.0
PA-active antibiotic prophylaxis within 3 months prior PABSI		28 (56)	19 (49)	9 (82)	0.085
Resistance	Carbapenems	17 (34)	11 (28)	6 (54)	0.15
	Piperacillin/ Tazobactam	16 (32)	9 (23)	7 (64)	**0.024**
	Fluoroquinolones	11 (22)	5 (13)	6 (54)	**0.008**
	Cephalosporins	12 (24)	5 (13)	7 (64)	**0.002**
	MDR	10 (20)	4 (10)	6 (54)	**0.004**
	Aminoglycosides	4 (8)	1 (3)	3 (27)	**0.029**

PA: *P. aeruginosa*

^1^ Chi-Square-Test or Fisher´s exact Test

The association between fluoroquinolone exposure (FQ-E) and fluoroquinolone resistance was at the margin of statistical significance, while the associations between either FQ-E and piperacillin/tazobactam resistance (PT-R) or FQ-E and cephalosporin resistance (Ceph-R) were statistically significant. Furthermore, previous therapies with carbapenems were found to be associated with carbapenem resistance. As there were no cases with cephalosporin-exposure, no associations for co-resistance could be statistically determined (Table 3).

**Table 3:** Regression analysis resistance and antibiotic pre-exposure

	Unadjusted OR (95%CI)	Univariate *p*-value	Unadjusted OR (95%CI)	Univariate *p*-value	Unadjusted OR (95%CI)	Univariate *p*-value	Unadjusted OR (95%CI)	Univariate *p*-value
	CP-*R*		PT-*R*		FQ-*R*		Ceph-*R*	
**CP-E**	5.5 (1.53–19.86)	**< 0.01**	3.06 (0.89–10.48)	0.08	4.76 (1.09–20.91)	**0.04**	1.38 (0.37–5.06)	0.63
**PT-E**	0.95 (0.29–3.11)	0.93	1.62 (0.49–5.36)	0.433	1.20 (0.31–4.61)	0.79	1.53 (0.42–5.66)	0.52
**FQ-E**	1.19 (0.35–4.02)	0.78	7.0 (1.38–35.62)	**0.02**	8.57 (0.99–73.58)	**0.05**	9.90 (1.16–84.47)	**0.04**
Ceph-E*	n.a.	n.a.	n.a.	n.a.	n.a.	n.a.	n.a.	n.a.

CP-E = Carbapenem exposure, PT-E = Piperacillin/Tazobactam exposure, FQ-E = Fluoroquinolone exposure, Ceph-E = Cephalosporin exposure, CP-R = Carbapenem-resistance, PT-R = Piperacillin/Tazobactam-resistance, FQ-R = Fluoroquinolone-resistance, Ceph-R = Cephalosporin-resistance

* no patients were exposed to *Pseudomonas*-active cephalosporins (ceftazidime/cefepime) within three months prior to PABSI

**Table 4:** Co-resistance of PABSI isolates

No of isolates	Resistance to	Co-resistance with antibiotic *n* (%)				
		P/T	CP	Cipro	PA-Ceph	AG
16	**P/T**		10 **(63)**	6 (38)	9 **(56)**	2 (13)
17	**CP**	10 **(59)**		8 (47)	8 (47)	4 (24)
11	**Cipro**	7 **(64)**	8 **(73)**		7 **(64)**	3 (28)
12	**PA-Ceph **	10 **(83)**	8 **(67)**	7 **(58)**		3 (25)
4	**AG**	2 (50)	4 **(100)**	3 **(75)**	3 **(75)**	

P/T = Piperacillin/Tazobactam; CP = Carbapenem (Imipenem or Meropenem); Cipro = Ciprofloxacin; PA-Ceph = *Pseudomonas*-active Cephalosporins (Ceftazidime or Cefepime); AG = Aminoglycosides

Co-resistance rates of > 50% are marked in bold

PA: *P. aeruginosa*

The PABSI isolates in our study showed a significant rate of co-resistance. PT-R isolates were often also carbapenem or cephalosporin resistant (63% and 56%, respectively). CP-R isolates were often also PT-R. FQ-R isolates were also predominantly carbapenem-, piperacillin/tazobactam, or cephalosporin-resistant (73%, 64%, and 64%, respectively). Aminoglycoside resistance (AS-R) was rare, with only 4 (8%) of isolates tested resistant. Conversely, when aminoglycoside resistance was present, CP-R, FQ-R and Ceph-R was also present in 100%, 75% and 75%, respectively (Table 4). A length of stay of more than 21 days prior to the onset of BSI was associated with a significantly higher risk of MDR-PABSI (p = < 0.01, data not shown), and mortality (Table 2).

**Fig. 1:**
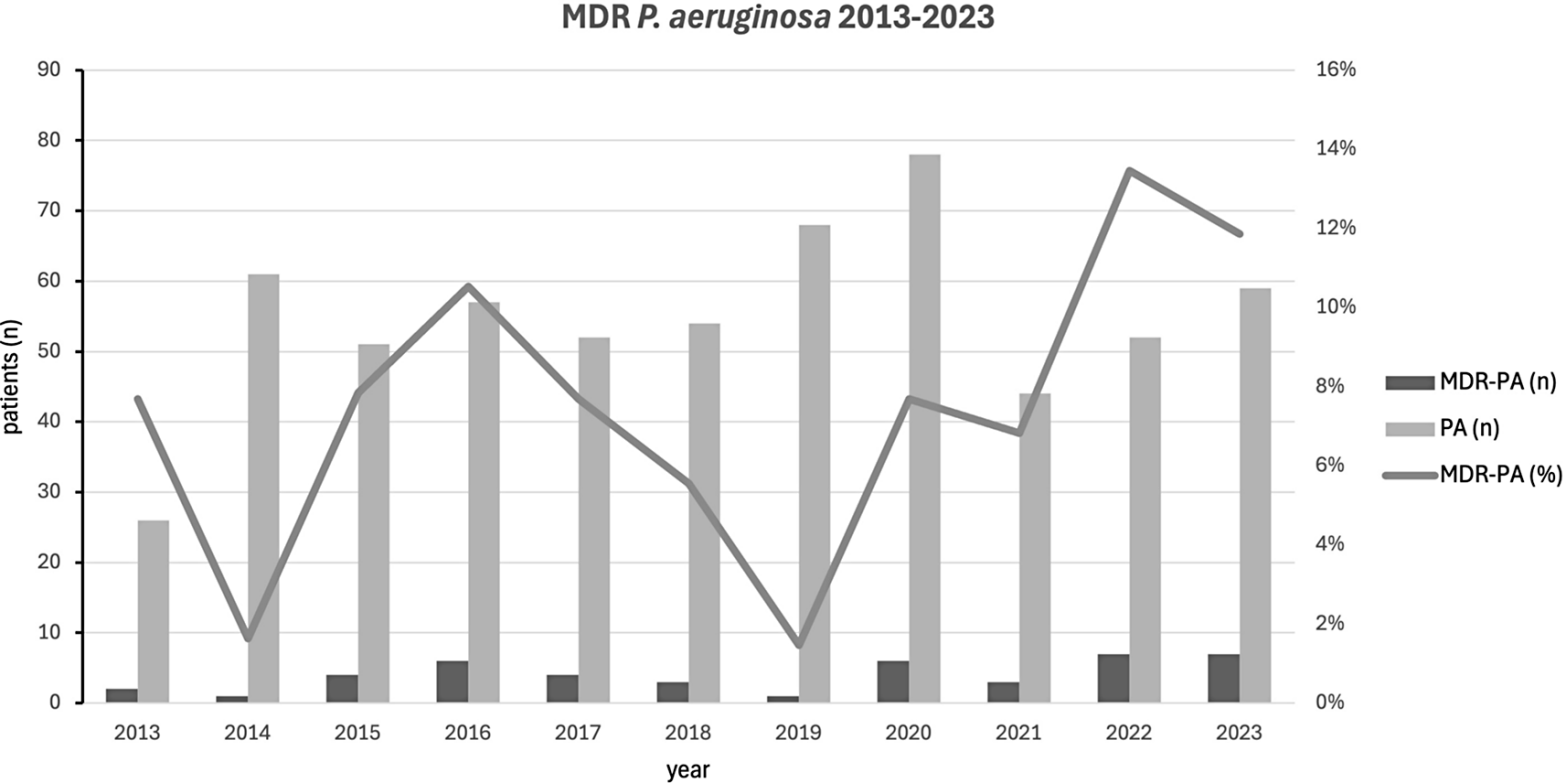
Local epidemiology of MDR *P. aeruginosa* during the observation period. MDR-PA; multidrug-resistant *P. aeruginosa*

During the observation period, the MDR rate among hematology wards was variable over the years and averaged at 7.5%, with an increase in the rate in 2022 and 2023 (Fig. 1).

In the univariate regression analysis, FQ-R, PT-R, Ceph-R, AG-R and MDR, but not CP-R were found to be associated with increased 30-day mortality (Table 5). Since there were no survivors among patients with intraabdominal infections, regression analysis could not be performed. The same applies to cases with septic shock; as there were no cases in the group of survivors. Taken together, cases with internal organ infection (lung or intraabdominal infection) exhibited a significantly elevated risk of fatal outcome, as in case of patients who did not receive adequate therapy within 24 h after the onset of fever. In the multivariable analysis, FQ-R and lack of adequate therapy within 24 h were significantly associated with unfavorable outcome (Table 5).

**Table 5:** Risk factors of fatal outcome at day 30 in PABSI by univariate and multivariate regression analysis

Characteristics	Unadjusted OR (95%CI)	Univariate *p*-value	OR	Multivariate *p*-value
Inappropriate antibiotic therapy within 24 h	6.78 (1.58-29.0)	**0.01**	**5.07 (1.07–24.07)**	**0.04**
Internal organ infection (Pneumonia or cIAI)	0.07 (0.01–0.33)	**< 0.001**		
Piperacillin/Tazobactam resistance	0.17 (0.041–0.721)	**0.02**		
Carbapenem-resistance	0.33 (0.08–1.30)	0.11		
Fluoroquinolone-resistance	0.12 (0.03–0.56)	**< 0.01**	**0.17 (0.03–0.83)**	**0.03**
Cephalosporin-resistance	0.08 (0.02–0.39)	**< 0.01**		
Aminoglycoside-resistance	0.07 (0.01–0.76)	**0.03**		
Multidrug-resistance	0.10 (0.02–0.46)	**< 0.01**		

## Discussion

In this monocentric retrospective study, we analyzed the risk factors and outcomes of adult hematological patients with BSI due to *P. aeruginosa* (PABSI) over nearly ten years. We found that antibiotic drug resistance is a main risk factor for increased mortality, most likely due to inadequate empirical therapy. The inadequacy of antibiotic therapy and the development of antibiotic resistance have been identified as risk factors for a fatal outcome in neutropenic patients with sepsis in previous studies [19]. These observations are consistent with those of other studies about PABSI caused by multidrug-resistant, sometimes hypervirulent isolates of *P. aeruginosa* in hematologic patients [20–22]. BSIs due to *P. aeruginosa* are associated with high rates of morbidity and mortality, with estimated mortality rates of 21–38% [6], which is in line with the observed mortality rate of 22% in our cohort [2, 19, 23]. Mortality is significantly higher in the presence of septic shock, in our cohort it was 100%, while another multicentric study showed a mortality rate of 75% [19]. We could confirm septic shock as a risk factor for mortality as demonstrated before [19]. In our cohort, as in other studies, previous antibiotic exposure was associated with occurrence of resistant *P. aeruginosa* [20, 24]. Of note, carbapenem exposure was associated with both carbapenem-resistance and fluoroquinolone-resistance in *P. aeruginosa.* Fluoroquinolone exposure was associated with both, cephalosporin-resistance and piperacillin/tazobactam resistance, whereas the association with fluoroquinolone-resistance remained at the margin of significance. We found higher rates of co-resistance compared to other studies [25]. *P. aeruginosa* has numerous intrinsic resistance mechanisms such as porin loss, efflux pumps and inactivating enzymes. It can also acquire resistance mechanisms, e.g. through mutation or horizontal gene transfer (e.g. metallo-betalactamases) [26]. The mechanism cannot be deduced from the resistance phenotype and since our strains were not available for whole genome sequencing, the causes for the observed resistance remain speculative.

It has been demonstrated that in patients with leukemia, the pathogen responsible for the BSI frequently dominates or colonizes the microbiota of the gastrointestinal (GI) tract [27]. In our hematology ward screening for MDR microorganisms is part of the institutional protocol. Conversely, preventing colonization, especially by MDR *P. aeruginosa*, could potentially reduce the risk of invasive infection. This could be achieved through antibiotic stewardship (AMS) measures to reduce selection pressure, supported by good hygienemanagement. AMS can help to reduce antibiotic exposure, e.g. by following guidelines, targeted therapy changes, shortening the duration of therapy and reducing fluoroquinolone prophylaxis (FQP) to a few high-risk constellations.

As about 60% of leukemia patients develop neutropenic fever and the prompt initiation of an empirical and *Pseudomonas*-active therapy, also depending on the local epidemiology, is strongly recommended. From 2013 to 2019, 308 BSI episodes occurred in hematological patients in our hospital. Neutropenic fever was present in 209 cases (68%). Gram-positive pathogens were detected in 53%, 41% of which were coagulase-negative staphylococci, corresponding to 22% of total detections. Gram-negative pathogens were identified in 47% of the cases, 12% of which were *P. aeruginosa*, corresponding to 6% of the total number of cases [28].

During 2013–2023 the rate of blood cultures positive for gram-positive organisms per year ranged from 54 to 75.2%, that of gram-negative organisms from 17,6% to 35,9%. The rate of *P. aeruginosa* positive blood cultures per year ranged from 1.7% to 9,8% corresponding finally to 50 patients having *P. aeruginosa* BSI. Of note, frequency of MDR *P. aeruginosa* at our hematology ward increased in the last years.

Most patients are extensively exposed to antibiotics and antibiotic selection pressure is a relevant problem [4, 29]. To minimize selection pressure and damage to the microbiota, antibiotic therapies should be prescribed for as long as necessary, but as short as possible. By deciding in favor of monotherapy versus combination therapy and a rational duration of treatment, antibiotic usage and thus selection pressure can be reduced. The superiority of combination therapy over monotherapy in PABSI is a subject of ongoing controversy. A meta-analysis showed no difference in terms of mortality, although data on infections with antibiotic resistant *P. aeruginosa*-BSI are scarce. For this reason, the current ESCMID guidelines do not include a recommendation for or against targeted combination therapy for infections caused by carbapenem-resistant *P. aeruginosa* [30]. In severe infections (sepsis), a combination is recommended, if monotherapy is based on either fosfomycin, a polymyxin or an aminoglycoside [30]. In line with this, the IDSA does not recommend combination therapy for PABSI, if one of the new beta-lactam/beta-lactamase inhibitor combinations (ceftazidime/avibactam, ceftolozane/tazobactam, imipenem/relebactam) or cefiderocol have been tested susceptible [31]. Current AGIHO guidelines strongly recommend the use of single-agent broad-spectrum *Pseudomonas*-active antibiotics such as piperacillin/tazobactam, ceftazidime, cefepime, meropenem or imipenem/cilastatin as first line antibiotic therapy [32]. However, in our cohort, 32% of patients received an empirical combination therapy, due to local treatment policy. With regard to the duration of therapy, it has been shown that the duration of treatment for febrile neutropenia, which is often determined by the time of regeneration, can be shortened in view of a 72 h fever-free status and clinical response, without any negative impact on clinical outcome [33]. Furthermore, a multicenter-study in onco-hematology patients with PABSI showed, that short-courses (7–11 days) of therapy were non-inferior in terms of clinical outcomes compared to prolonged courses (12-21days) [34].

The benefits of FQP have also been the subject of controversial debate for years. In the meantime, it has been repeatedly demonstrated that, although the omission of an FQP is associated with an increase in BSI, it is not associated with increased mortality [35, 36].

The effects of FQP on resistance selection were shown not only for *P. aeruginosa* infections, but also for both viridans *streptococci* and *Enterobacterales* [37] which in turn leads to reduced effectiveness of prophylaxis [38]. The empirical use of fluoroquinolones for combination therapy in neutropenic patients with sepsis can therefore not be recommended in centers where FQP is widely used or if colonization with a FQ-R pathogen such as *P. aeruginosa* or *Enterobacterales* is documented.

These observations compelled other centers to leave the FQP [39, 40]. Discontinuation of FQP has been shown to decrease FQ-R and 3rd generation cephalosporin resistance due to ESBL-producing *Enterobacterales*, without the increase of serious infectious complications [41].

At our center, FQP has been temporarily discontinued in the stem cell unit but is still in place in patients undergoing intensive chemotherapy for acute leukemia. As a result, potentially resistant pathogens, including *P. aeruginosa*, may be already selected prior to stem cell transplantation.

As previously shown, FQP, prior antibiotic therapy with either piperacillin/tazobactam or a *Pseudomonas*-carbapenem, and hematological disease are risk factors for BSI with MDR *P. aeruginosa*.

Antibiotic stewardship in hematology may therefore contribute to reducing infections caused by multi-resistant pathogens and the maintenance of a protective microbiota, thereby improving patient outcomes [42].

As a consequence of our study, we would like to strengthen antibiotic stewardship efforts on hematology wards. In the light of the observed increase in mortality associated with FQR, the use of FQP in patients with acute leukemia under intensive chemotherapy should be reconsidered.

## Limitations

It should be noted that this study has some limitations. First, this is a retrospective study, which means that not all relevant data was available. Second, the evaluation is monocentric, and BSIs with *P. aeruginosa* are not very frequent, resulting in a relatively low number of cases, with only 50 patients included. During the 10-year period, some treatment standards have changed, e.g. FQP in the stem cell transplant unit. Furthermore, some new antibiotics became available and colistin-based regimen were no longer necessary in the calculated treatment of bloodstream infections due to MDR gram-negative pathogens.

Consequently, our conclusions need to be tested in large, multicenter and potentially interventional studies.

## Data Availability

No datasets were generated or analyzed during the current study.
